# Author Correction: An atlas of epithelial cell states and plasticity in lung adenocarcinoma

**DOI:** 10.1038/s41586-024-07277-4

**Published:** 2024-03-18

**Authors:** Guangchun Han, Ansam Sinjab, Zahraa Rahal, Anne M. Lynch, Warapen Treekitkarnmongkol, Yuejiang Liu, Alejandra G. Serrano, Jiping Feng, Ke Liang, Khaja Khan, Wei Lu, Sharia D. Hernandez, Yunhe Liu, Xuanye Cao, Enyu Dai, Guangsheng Pei, Jian Hu, Camille Abaya, Lorena I. Gomez-Bolanos, Fuduan Peng, Minyue Chen, Edwin R. Parra, Tina Cascone, Boris Sepesi, Seyed Javad Moghaddam, Paul Scheet, Marcelo V. Negrao, John V. Heymach, Mingyao Li, Steven M. Dubinett, Christopher S. Stevenson, Avrum E. Spira, Junya Fujimoto, Luisa M. Solis, Ignacio I. Wistuba, Jichao Chen, Linghua Wang, Humam Kadara

**Affiliations:** 1 Department of Genomic Medicine, The University of Texas MD Anderson Cancer Center, Houston, TX USA; 2 Department of Translational Molecular Pathology, The University of Texas MD Anderson Cancer Center, Houston, TX USA; 3 Department of Pulmonary Medicine, The University of Texas MD Anderson Cancer Center, Houston, TX USA; 4 Graduate Program in Developmental Biology, Baylor College of Medicine, Houston, TX USA; 5 The University of Texas Health Houston Graduate School of Biomedical Sciences, Houston, TX USA; 6 Department of Biostatistics, Epidemiology and Informatics, Perelman School of Medicine, University of Pennsylvania, Philadelphia, PA USA; 7 Department of Thoracic, Head and Neck Medical Oncology, The University of Texas MD Anderson Cancer Center, Houston, TX USA; 8 Department of Cardiovascular and Thoracic Surgery, The University of Texas MD Anderson Cancer Center, Houston, TX USA; 9 Department of Epidemiology, The University of Texas MD Anderson Cancer Center, Houston, TX USA; 10 Department of Medicine, The University of California Los Angeles, Los Angeles, CA USA; 11 Lung Cancer Initiative at Johnson & Johnson, Boston, MA USA; 12 Section of Computational Biomedicine, School of Medicine, Boston University, Boston, MA USA

**Keywords:** Cancer genomics, Non-small-cell lung cancer

Correction to: *Nature* 10.1038/s41586-024-07113-9 Published online 28 February 2024

In the version of the article initially published, the colours used in the top right panel of Fig. 4a did not match the colour-blind-friendly palette used in that panel’s key. The figure has now been updated in the HTML and PDF versions of the article.

