# Supplementary material for: An atlas of epithelial cell states and plasticity in lung adenocarcinoma
Source: Nature. 2024 Feb 28;627(8004):656–63. doi: 10.1038/s41586-024-07113-9 (PMC10954546; doi:10.1038/s41586-024-07113-9)
Supplement: Supplementary file 2 — Reporting Summary [file 41586_2024_7113_MOESM2_ESM.pdf]

nature portfolio

Corresponding author(s): Humam Kadara, Linghua Wang, Jichao Chen

Last updated by author(s): Feb 13, 2024

Reporting Summary

Nature Portfolio wishes to improve the reproducibility of the work that we publish. This form provides structure for consistency and transparency in reporting. For further information on Nature Portfolio policies, see our Editorial Policies and the Editorial Policy Checklist.

Statistics

For all statistical analyses, confirm that the following items are present in the figure legend, table legend, main text, or Methods section.

n/a	Confirmed	
☐	☒	The exact sample size (*n*) for each experimental group/condition, given as a discrete number and unit of measurement
☐	☒	A statement on whether measurements were taken from distinct samples or whether the same sample was measured repeatedly
☐	☒	The statistical test(s) used AND whether they are one- or two-sided<br>*Only common tests should be described solely by name; describe more complex techniques in the Methods section.*
☐	☒	A description of all covariates tested
☐	☒	A description of any assumptions or corrections, such as tests of normality and adjustment for multiple comparisons
☐	☒	A full description of the statistical parameters including central tendency (e.g. means) or other basic estimates (e.g. regression coefficient) AND variation (e.g. standard deviation) or associated estimates of uncertainty (e.g. confidence intervals)
☐	☒	For null hypothesis testing, the test statistic (e.g. *F*, *t*, *r*) with confidence intervals, effect sizes, degrees of freedom and *P* value noted<br>*Give P values as exact values whenever suitable.*
☒	☐	For Bayesian analysis, information on the choice of priors and Markov chain Monte Carlo settings
☐	☒	For hierarchical and complex designs, identification of the appropriate level for tests and full reporting of outcomes
☐	☒	Estimates of effect sizes (e.g. Cohen's *d*, Pearson's *r*), indicating how they were calculated

Our web collection on statistics for biologists contains articles on many of the points above.

Software and code

Policy information about availability of computer code

Data collection

A detailed description of patient cohorts, clinical characteristics, data collection and filtering is included in the Methods.
All data from single cell RNA sequencing and spatial transcriptomics analysis of human and mouse lung tissues were generated in-house in laboratory of Dr. Humam Kadara at MD Anderson Cancer Center and as described in the Methods section.
Cell Ranger Single Cell Software Suite (version 3.0.1) was used to process raw scRNA-seq data.
Spaceranger was used for analysis of ST data (version 1.3.0 for human ST data and version 2.0.0 for mouse ST data).
ImageScope software (Leica Microsystems, Inc.) was used to visualize H&E stained slides.
HALO software (Indica Labs, Albuquerque, NM) was used for pathological annotation of tissue slides.
Imaris software was used to analyze IF images obtained with confocal microscopy.
Data was recorded using Microsoft Excel v.2016.
Codes for analysis of scRNA-seq, WES, and ST data are available at Zenodo (https://doi.org/10.5281/zenodo.8280138) and GitHub (https://github.com/guangchunhan/LUAD_Code).

Data analysis

Single-cell RNA-seq data processing and quality control
Raw scRNA-seq data were pre-processed (demultiplex cellular barcodes, read alignment and generation of gene count matrix) using CellRanger Single Cell Software Suite (v.3.0.1) provided by 10X Genomics. For read alignment of human and mouse scRNA-seq data, human reference GRCh38 (hg38) and mouse reference GRCm38 (mm10) genomes were used, respectively. Detailed quality control metrics were generated and evaluated, and cells were carefully and rigorously filtered to obtain high-quality data for downstream analyses. In brief, for basic quality filtering, cells with low-complexity libraries (in which detected transcripts were aligned to <200 genes such as cell debris, empty drops and low-quality cells) were filtered out and excluded from subsequent analyses. Probable dying or apoptotic cells in which > 15% of transcripts derived from the mitochondrial genome were also excluded. For scRNA-seq analysis of Gprc5a-/-;SftpcCreER/+;RosaSun1GFP/+ mice, cells mice, cells with more than 500 detected genes or with a mitochondrial gene fraction that is greater or equal to 15% were filtered out using Seurat.

Doublet detection and removal, and batch effect evaluation and correction

Probable doublets or multiplets were identified and carefully removed through a multi-step approach as described in previous studies. In brief, doublets or multiplets were identified based on library complexity, whereby cells with high-complexity libraries in which detected transcripts are aligned to >6,500 genes were removed and, based on cluster distribution and marker gene expression,whereby doublets or multiplets forming distinct clusters with hybrid expression features and/or exhibiting an aberrantly high gene count were also removed. Expression levels and proportions of canonical lineage-related marker genes in each identified cluster were carefully reviewed. Clusters co-expressing discrepant lineage markers were identified and removed. Doublets or multiplets were also identified using the doublet detection algorithm DoubletFinder. The proportion of expected doublets was estimated based on cell counts obtained before scRNA-seq library construction. Data normalization was then performed using Seurat. on the filtered gene–cellmatrix. Statistical assessment of possible batch effects was performed on non-malignant epithelial cells using the R package ROGUE, an entropy-based statistic, as described in previous studies, and Harmony was run with default parameters to remove batcheffects present in the PCA space.

Unsupervised clustering and subclustering analysis
The function FindVariableFeatures of Seurat was applied to identify highly variable genes for unsupervised cell clustering. PCA was performed on the top 2,000 highly variable genes. The elbow plot was generated with the ElbowPlot function of Seurat and, based on which, the number of significant principal components (PCs) was determined. The FindNeighbors function of Seurat was used to construct the shared nearest neighbour (SNN) graph based on unsupervised clustering performed using the Seurat function FindClusters. Multiple rounds of clustering and subclustering analyses were performed to identify major epithelial cell types and distinct cell transcriptional states. Dimensionality reduction and 2D visualization of cell clusters was performed using UMAP and the Seurat function RunUMAP. The number of PCs used to calculate the embedding was the same as that used for clustering. For analysis of human epithelial cells, ROGUE was used to quantify cellular transcriptional heterogeneity of each cluster. Subclustering analysis was then performed for low-purity clusters identified by ROGUE. Hierarchical clustering of major epithelial subsets was performed on the Harmony batch-corrected PCA dimension reduction space. For malignant cells, except for global UMAP visualization, downstream analyses, including identification of large-scale CNVs, inference of cancer cell differentiation states, quantification of meta-program expression, trajectory analysis and mutation analysis, were performed without Harmony batch correction. The hierarchical tree of human epithelial cell lineages was computed based on Euclidean distance using the Ward linkage method, and the dendrogram was generated using the R function plot.hc. For scRNA-seq analysis of Gprc5a-/- mice, the top-ranked ten PCs were selected using the elbowplot function. SNN graph construction was performed with resolution parameter = 0.4, and UMAP visualization was performed with default parameters. For scRNA-seq analysis of Gprc5a-/-;SftpcCreER/+;RosaSun1GFP/+ mice, the top-ranked 20 Harmony-corrected PCs were used for SNN graph construction, and unsupervised clustering was performed with resolution parameter = 0.4. UMAP visualization was performed with the RunUMAP function with min.dist = 0.1. Differentially expressed genes (DEGs) of clusters were identified using the FindAllMarkers function with FDR-adjusted P value < 0.05 and log (fold change) > 1.2.

Identification of malignant cells and mapping KRAS codon 12 mutations
Malignant cells were distinguished from non-malignant subsets based on information integrated from multiple sources as described in previous studies. The following strategies were used to identify malignant cells. (1) Cluster distribution: owing to the high degree of inter-patient tumour heterogeneity, malignant cells often exhibit distinct cluster distribution compared with normal epithelial cells. Although non-malignant cells derived from different patients are often clustered together by cell type, malignant cells from different patients probably form separate clusters. (2) CNVs: we applied inferCNV (v.1.3.2) to infer large-scale CNVs in each individual cell with T cells as the reference control. To quantify CNVs at the cell level, CNV scores were aggregated using a previously described strategy. In brief, arm-level CNV scores were computed based on the mean of the squares of CNV values across each chromosomal arm. Arm-level CNV scores were further aggregated across all chromosomal arms by calculating the arithmetic mean value of the arm-level scores using the R function mean. (3) Marker gene expression: expression of lung epithelial lineage-specific genes and LUAD-related oncogenes was determined in epithelial cell clusters. (4) Cell-level expression of KRASG12D mutations: as we previously described, BAM files were queried for KRASG12D mutant alleles, which were then mapped to specific cells. KRASG12D mutations,along with cluster distribution, marker gene expression and inferred CNVs as described above, were used to distinguish malignant cells from non-malignant cells. Following clustering of malignant cells from all patients, an absence of malignant cells that were identified from P12 or P16 was noted. This can be possibly attributed to the low number of epithelial cells captured in tumour samples from these patients (Supplementary Table 2).

Mapping KRAS codon 12 mutations
To map somatic KRASG12D mutations at single-cell resolution, alignment records were extracted from the corresponding BAM files using mutation location information. Unique mapping alignments (MAPQ = 255) labelled as either PCR duplication or secondary mapping were filtered out. The resulting somatic variant carrying reads were evaluated using Integrative Genomics Viewer (IGV) and the CBtags were used to identify cell identities of mutation-carrying reads. To estimate the VAF of KRASG12D mutation and cell fraction of KRASG12D-carrying cells within malignant and non-malignant epithelial cell subpopulations (for example, malignant cells from all LUADs, malignant cells from KM-LUADs, KACs from KM-LUADs), reads were first extracted based on their unique cell barcodes and BAM files were generated for each subpopulation using samtools (v.1.15). Mutations were then visualized using IGV, and VAFs were calculated by dividing the number of KRASG12D -carrying reads by the total number of uniquely aligned reads for each subpopulation. A similar approach was used to visualize KRASG12C-carrying reads and to calculate the VAF of KRASG12C in KACs of normal tissues from KM-LUAD cases. To calculate the mutation-carrying cell fraction, extracted reads were mapped to the KRASG12D locus from BAM files using AlignmentFile and fetch functions in pysam package. Extracted reads were further filtered using the'Duplicate' and 'Quality' tags to remove PCR duplicates and low-quality mappings. The number of reads with or without KRASG12D mutation in each cell was summarized using the CB tag in read barcodes. Mutation-carrying cell fractions were then calculated as the ratio of the number of cells with at least one KRASG12D read over the number of cells with at least one high-quality read mapped to the locus.

PCA analysis of malignant cells and quantification of transcriptome similarity
Raw unique molecular identifier counts of identified malignant cells were log-normalized and used for PCA analysis using Seurat (RunPCA function). PCA dimension reduction data were extracted using the Embeddings function. The top three most highly ranked PCs were exported for visualization using JMP (v.15). 3D scatterplots of PCA data were generated using the scatterplot 3D tool in JMP (v.15). Bhattacharyya distances were calculated using the bhattacharyya.dist function from the R package fpc (v.2.2-9). The top 25 highly ranked PCs were used for both patient-level and cell-level distance calculations. For Bhattacharyya distance quantification at the cell level,100 cells were randomly sampled for each patient group defined by driver mutations (for example, KM-LUADs). The random sampling process was repeated 100 times, and pairwise Bhattacharyya distances were then calculated between patient groups. Differences in Bhattacharyya distances between patient groups were tested using Wilcoxon rank-sum tests, and boxplots were generated using the geom_boxplot function from the R package ggplot2 (v.3.2.0).

Determination of non-malignant cell types and states
Non-malignant cell types and states were determined based on unsupervised clustering analysis following batch effect correction using

Harmony. Two rounds of clustering analysis were performed on non-malignant cells to identify major cell types and cell transcriptional states within major cell types. Clustering and UMAP visualization of human normal epithelial cells (Extended Data Fig. 1a) were performed using Seurat with default parameters. Specifically, the parameters k.param = 20 and resolution = 0.4 were used forSNN graph construction and cluster identification, respectively. UMAP visualization was performed with default parameters (min.dist = 0.3). For clustering analysis of airway and alveolar epithelial cells, the RunPCA function was used to determine the most contributing top PCs for each subpopulation and similar clustering parameters (k.param = 20 and resolution = 0.4) were used for SNN graph construction and cluster identification. UMAP plots were generated with min.dist = 0.3 using the RunUMAP function in Seurat. Density plots of alveolar intermediate cells were generated using the stat_densit_2d function in the R package ggplot2 (v.3.3.5) with the first two UMAP dimension reduction data as the input. DEGs for each cluster were identified using the FindMarkers function in Seurat with a FDR-adjusted P < 0.05 and a fold change cut-off > 1.2. Canonical epithelial marker genes from previously published studies by our group and others were used to annotate normal epithelial cell types and states. Bubble plots were generated for select DEGs and canonical markers to define AT1 cells (AGER1+ ETV5+ PDPN+), AT2 cells (SFTPB, SFTPC, ETV5+), SCGB1A1+SFTPC+ dual-positive cells, AICs (AGER1+ETV5+PDPN+ and SFTPB+SFTPC+), club and secretory cells (SCGB1A1 +SCGB3A1+CYP2F1+), basal cells (KRT5+TP63+), ciliated cells (CAPS+PIFO+FOXJ1+), ionocytes (ASCL3+FOXI+), neuroendocrine cells (CALCA +ASCL1+) and tuft cells (ASCL2+MGST2+PTGS1+). KACs were identified by unsupervised clustering of AICs and defined based on previously reported marker genes, including significant upregulation of the following genes relative to other alveolar cells: KRT8, CLDN4, PLAUR, CDKN1A and CDKN2A.

Scoring of curated gene signatures
Genes in previously defined ITH MPs were downloaded from the original study. Among a total of 41 consensus ITH MPs identified, MPs with unassigned functional annotations (unassigned MPs 38–41; n = 4), neural and haematopoietic lineage-specific MPs (MPs 25–29, MPs 33–37; n = 10) and cell-type-specific MPs irrelevant to LUAD (MPs 22–24 secreted/cilia, MP 32 skin-pigmentation; n = 4) were filtered out, resulting in 23 MPs that closely correlated with hallmarks of cancer and which were used for further analysis. Signature scores were computed using the AddModuleScore function in Seurat as previously described. The KRAS signature used in this study was derived by calculating DEGs between the KRAS mutant malignant-cell-enriched cluster and other malignant cells (FDR-adjusted P value < 0.05, log(fold change) > 1.2; Extended Data Fig. 2i). Human and mouse KAC signatures and the human 'other AIC'signature were derived by calculating DEGs using FindAllMarkers among alveolar cells (FDR-adjusted P value < 0.05, log(fold change) > 1.2). Mouse genes in the p53 pathway were downloaded from the Molecular Signature Database (MSigDB; https://www.gsea-msigdb.org/gsea/msigdb/mouse/geneset/HALLMARK_P53_PATHWAY; MM3896). Signature scores for KACs, other AICs, KRAS andp53 were calculated using the AddModuleScore function in Seurat.

Analysis of alveolar cell differentiation states and trajectories
Analysis of differentiation trajectories of lung alveolar and malignant cells was performed using Monocle 2 by inferring the pseudotemporal ordering of cells according to their transcriptome similarity. Monocle 2 analysis of malignant cells from P14 was performed using default parameters with the detectGenes function. Detected genes were further required to be expressed by at least 50 cells. For construction of the differentiation trajectory of lineage-labelled epithelial cells (GFP), the top 150 DEGs (FDR-adjusted P value < 0.05, log(fold change) > 1.5, expressed in 50 cells or more) ranked by fold-change of each cell population from NNK-treated samples were used for ordering cells with the setOrderingFilter function. Trajectories were generated using the reduceDimension function with the method set to 'DDRTree'. Trajectory roots were selected based on the following criteria: (1) inferred pseudotemporal gradient; (2) CytoTRACE score prediction; and (3) careful manual review of the DEGs along the trajectory. To depict expression dynamics of ITH MPs, ITH MP scores were plotted along the pseudotime axis and smoothed lines were generated using the smoother tool in JMP Pro(v.15). Using the raw counts without normalization as input, CytoTRACE was applied with default parameters to infer cellular differentiation states to complement trajectory analysis and further understand cellular differentiation hierarchies. The normalmixEM function from the R package mixtools was used to determine the CytoTRACE score threshold in AICs with k = 2. A final threshold of 0.58was used to dichotomize AICs into high-differentiation and low-differentiation groups. The Wasserstein distance metric was applied usingR package transport (v.0.13) to quantify the variability of distribution of CytoTRACE scores. The function wasserstein1d was used tocalculate the distance between the distribution of actual CytoTRACE scores of one patient and the distribution of simulated data with identical mean and standard deviation. The robustness of Monocle 2-based pseudotemporal ordering prediction was validated by independent pseudotime prediction tools including Palantir, Slingshot and Cellrank. Slingshot (v.2.6.0) pseudotime prediction was performed using slingshot function with reduceDim parameter set to 'PCA' and other parameters set to defaults. Cellrank prediction was performed using the CytoTRACEKernel function with default parameters from Cellrank python package (v.1.5.1). Palantir prediction was performed using Palantir python package (v.1.0.1). A diffusion map was generated using run_diffusion_maps function withn_components = 5. Palantir prediction was generated using run_palantir function with num_waypoints = 500 and other parameters set to defaults. Inferred pseudotime by the three independent methods was then integrated with that generated by Monocle 2 for each single cell,followed by pairwise mapping and correlation analysis. Cell density plots were generated using Contour tool in JMP (v.15) with n = 10 gradient levels and contour type parameter set to 'Nonpar Density'. To assess the pseudotime prediction consistency between Monocle 2and the three independent methods, Spearman's correlation coefficients were calculated and statistically tested using cor.test function in R.

ST data generation and analysis
ST profiling of formalin fixation and paraffin-embedding (FFPE) tissues of P14 LUAD sample and of three lung tissues from two Gprc5a-/- mice was performed using the Visium platform from 10X Genomics according to the manufacturer's recommendations and as previously reported. P14 FFPE tissues were collected from areas adjacent to the tissues analysed by scRNA-seq. Regions of interest per tissue or sample,each comprising a 6.5 × 6.5 mm capture area, were selected based on careful annotation of H&E-stained slides that were digitally acquired using an Aperio ScanScope Turbo slide scanner (Leica Microsystems). HALO software (Indica Labs) was used for pathological annotation (tumour areas, blood vessels, bronchioles, lymphoid cell aggregates, macrophages, muscle tissue, normal parenchyma and reactive pneumocytes) of H&E histology images. Spot-level histopathological annotation and visualization was generated using loupebrowser (v.6.3.0). In brief, cloupe files generated from Space Ranger were loaded into the loupe browser. Visualization of annotation was then generated in svg formats using the export plot tool. ST RNA-seq libraries were generated according to the manufacturer'sinstructions, each with up to about 3,600 uniquely barcoded spots. Libraries were sequenced on an Illumina NovaSeq 6000 platform to achieve a depth of at least 50,000 mean read pairs per spot and at least 2,000 median genes per spot.
Demultiplexed raw sequencing data were aligned, and gene level expression quantification was generated with analysis pipelines as previously described. In brief, demultiplexed clean reads were aligned against the UCSC human GRCh38 (hg38) or the GRCm38(mm10) mouse reference genomes by Spaceranger (v.1.3.0 for human ST data and v.2.0.0 for mouse ST data) and using default settings. Generated ST gene expression count matrices were then analysed using Seurat (v.4.1.0) to perform unsupervised clustering analysis. Using default parameters, the top-ranked 30 PCA components were used for SNN graph construction and clustering and for UMAP low-dimension space embedding with default parameters. UMAP analysis was performed using the RunUMAP function. The SpatialDimPlotfunction was used to visualize unsupervised clustering. The R package inferCNV was used for copy number analysis. Reference spots used in CNV analysis were selected on the basis of careful review of cluster marker genes using the DotPlot function from Seurat and inspection of pathological annotation. CNV scores were

calculated by computing the standard deviations of CNVs inferred across 22autosomes. Loupe browser (v.6.3.0) was used for visualization of pathological annotation results. Expression levels of genes of interest (for example, KRT8) as well as signatures of interest (for example, KAC and KRAS) were measured and directly annotated on histology images with pixel-level resolution using the TESLA (v.1.2.2) machine learning framework (https://github.com/jianhuupenn/TESLA). TESLA can compute superpixel-level gene expression and detect unique structures within and surrounding tumours by integrating information from high-resolution histology images. The annotation and visualize_annotation functions were used to annotate regions with high signature signals. KRT8, PLAUR, CLDN4, CDKN1A and CDKN2A were used for 'KAC markers' signature annotation in the human ST analysis. For mouse ST data, Krt8, Plaur, Cldn4, Cdkn1a and Cdkn2a were used for 'KAC signature' annotation. Gene level expression visualization of Krt8 and Plaur was generated using the scatter function from scanpy (v.1.9.1).
Deconvolution analysis was conducted using CytoSPACE (https://github.com/digitalcytometry/cytospace). Annotated scRNA-seq data were first transformed intoa compatible format using function generate_cytospace_from_scRNA_seurat_object. Visium spatial data were prepared using the functiongenerate_cytospace_from_ST_seurat_object. Deconvolution was performed using CytoSpace function (v.1.0.4) with default parameters. To determine neighbouring cell composition for a specific cell population in Visium data, CytoSPACE was first applied to annotate every spot with the most probable cell type. Neighbouring spots were defined as the six spots surrounding each spot and, accordingly, the neighbouring cell composition for specific cell types were computed. Trajectory construction of ST data was performed using Monocle 2 with the DDRTree method using DEGs with FDR-adjusted P value < 0.05.

Bulk DNA extraction and WES
Total DNA was isolated from homogenized cryosections of human lung tissues and, when available, from frozen peripheral blood mononuclear cells (PBMCs) using a Qiagen AllPrep mini kit (80204) or a DNeasy Blood and Tissue kit (69504), respectively (both from Qiagen) according to the manufacturer's recommendations. Qubit 4 Fluorometer (Thermo Fisher Scientific) was used for measurement of DNA yield. TWIST-WES was performed on a NovaSeq 6000 platform at a depth of 200x for tumour samples and 100x for NL and PBMCs to analyse recurrent driver mutations and using either PBMCs or distant NL tissues when blood draw was not consented, as germline control. WES data were processed and mapped to the human reference genome, and somatic mutations were identified and annotated as previously described with further filtration steps. In brief, only MuTect calls marked as 'KEEP' were selected and taken into the next step. Mutations with a low VAF (<0.02) or low alt allele read coverage (<4) were removed. Then, common variants reported by ExAc (the Exome Aggregation Consortium, http://exac.broadinstitute.org), Phase-3 1000 Genome Project (http://phase3browser.1000genomes.org/Homo_sapiens/Info/Index) or the NHLBI GO Exome Sequencing Project (ESP6500) (http://evs.gs.washington.edu/EVS/) with minor allele frequencies greater than 0.5% were further removed. Intronic mutations, mutations at 3' or 5' UTR or UTR-flanking regions, and silent mutations were also removed. The mutation load in each tumour was calculated as the number of nonsynonymous somatic mutations (nonsense, missense, splicing, stop gain, stop loss substitutions as well as frameshift insertions and deletions).

Survival analysis
Analysis of OS in the TCGA LUAD and PROSPECT cohorts was performed as previously described. KRAS mutation status in TCGA LUAD samples was downloaded from cBioPortal (https://www.cbioportal.org, study ID: luad_tcga_pan_can_atlas_2018). For TCGA dataset, clinical data were downloaded from the PanCanAtlas study. The logrank test and Kaplan–Meier methods were used to calculate P values between groups and to generate survival curves, respectively. Statistical significance testing for all survival analyses was two-sided. To control for multiple hypothesis testing, Benjamini–Hochberg method was applied to correct P values, and FDR q values were calculated where applicable. Results were considered significant at P value or FDR q value of <0.05. Multivariate survival analysis was performed using a Cox proportional hazards regression model that calculated the hazard ratio, the 95% confidence interval and P values when using pathologic stage, age, KAC and 'other AIC' signatures as covariables.

Analysis of public datasets
Publicly available datasets were obtained from the Gene Expression Omnibus (GEO) database (https://www.ncbi.nlm.nih.gov/geo/) under accession numbers GSE149813, GSE154989, GSE150263, GSE102511 and GSE219124. Details of the studies analysed are as follows: GSE149813 investigated single lung cells from KrasLSL-G12D;LSL-YFP mice with Ad5CMV-Cre infection; GSE154989 studied AT2 lineage-labelled cells from lungs of KrasLSL-G12D/+;Rosa26LSL-tdTomato/+ mice. Gene expression count matrices of dataset interrogating KrasG12D-driven mouse model from GSE149813 were pre-processed using Seurat following the same filtering steps in that original report. For the GSE154989 dataset, cells used for analysis were the ones labelled as "PASSED_QC" in supplementary table S7 in that study. For the GSE149813 dataset, cells with >500 median number of genes detected and <10% fraction of mitochondrial genome derived reads, and according to the pre-processing methods described in their original report, were retained for analysis. Cells with >7,500 number of genes detected were further filtered to remove potential doublets or multiplets, resulting in 8,304 cells in total for downstream analysis. Both datasets were integrated with mouse cell data generated in this study using Harmony with default parameters settings. The top ranked 20 Harmony-corrected PCs were used for clustering with the FindClusters function using resolution = 0.4. UMAP dimension reduction embedding was performed using the RunUMAP function with the same set of Harmony-corrected PCs. Gene expression levels and frequencies of representative cluster marker genes were visualized using DotPlot function from Seurat. The KAC signature score was calculated using the AddModuleScore function from Seurat. The mouse KAC signature was also studied in human AT2 cells with and without inducible KRASG12D (dataset GSE150263). Cell filtration criteria described in the original report were followed to filter out potential dead cells and doublets (number of detected genes > 800 and the percent of mitochondrial gene reads fraction < 25%). The 20top-ranked PCs were used for clustering using the FindClusters function with resolution = 0.1. UMAP dimension reduction embeddings were computed using the same SNN graph. The KAC signature score was calculated using AddModuleScore function from Seurat package.
The bulk RNA-seq dataset GSE102511 was a previously published dataset by our group and comprised normal lung tissues, precursor AAHs and matched LUADs (n = 15, each). The previously published bulk RNA-seq data GSE219124 were generated on cancer stem cell and stem cell-like progenitor cells, in the form of spheres, and their parental MDA-F471 counterparts (a cell line we had developed and cultured from a KM-LUAD of an NNK-exposed Gprc5a-/- mouse). To interrogate the association of KACs with tumour formation, gene expression matrices of bulk RNA-seq data GSE102511 (TPM count matrix) and GSE219124 (FPKM count matrix) were extracted and used for quantification of KAC signature expression using MCPcounter (v.1.2.0) R package. Heatmaps were generated using pheatmap (v.1.0.12) R package.
Mouse KACs from this study were compared to mouse Krt8 transitional cells involved in alveolar regeneration post-acute lung injury from a previous study. Overlapping marker genes between mouse KACs and the previously reported Krt8 transitional cells were statistically evaluated using the ggvenn (v.0.1.9) R package using the top-ranked 50 marker genes based on fold change from each study.

Histopathological and IF analysis of mouse lung tissues
Lungs of Gprc5a-/- mice (n= 2 per treatment and time point) were inflated with formalin by gravity drip inflation, excised, examined for lung surface lesions by macroscopic observation and processed for FFPE, sectioning and H&E staining. Stained slides were digitally scanned using an Aperio ScanScope Turbo slide scanner (Leica Microsystems) at ×200 magnification, and visualized using ImageScope software (Leica Microsystems). Unstained lung tissue sections were obtained for IF analysis of LAMP3 (clone 391005, Synaptic Systems), KRT8 (TROMA-I clone from the University of Iowa DSHB) and PDPN (clone 8.1.1, from the University of Iowa DSHB). Lung FFPE tissue samples were obtained in the

same manner from Gprc5a-/-;SftpcCreER/+;RosaSun1GFP/+ mice at 3 months after exposure to saline or NNK (n = 2 mice per condition) and following injection with tamoxifen.
Tissue sections were obtained for H&E staining and assessment of tumour development, and unstained sections were used for IF analysis using antibodies against GFP (AB13970, Abcam, 1:5000), LAMP3 (391005, Synaptic Systems, 1:10,000), KRT8 (TROMA-I,University of Iowa Developmental Studies Hybridoma Bank, 1:100), PDPN (clone 8.1.1, University of Iowa Developmental Studies Hybridoma Bank, 1:100), claudin 4 (ZMD.306, Invitrogen, 1:250), and PRKCDBP (cavin 3, Proteintech, 1:250). Slides werethen stained with fluorophore-conjugated secondary antibodies and 4',6'-diamidino-2-phenylindole (DAPI). Sections were mounted with Aquapolymount (18606, Polysciences), cover slipped, imaged using an Andor Revolution XDi WD spinning disk confocal microscope and analysed using Imaris software (Oxford Instruments).
Formalin-inflated lung lobes from Krt8-creER;RosatdT/+ mice were cryoprotected in 20% sucrose in PBS containing 10% OCT compound (4583, Tissue-Tek) overnight on a rocker at 4 °C and embedded in OCT. The next day, 10 micrometer cryosections were blocked in PBS with 0.3% Triton X-100 and 5% normal donkey serum (017-000-121, Jackson ImmunoResearch) and incubated overnight in a humidified chamber at4 °C with primary antibodies diluted in PBS with 0.3% Triton X-100 and raised against NKX2-1 (sc-13040, Santa Cruz, 1:1000),LAMP3 (same as above) and KRT8 (same as above). The next morning, sections were washed followed by incubation with secondary antibodies (Jackson ImmunoResearch) and DAPI. Slides were then washed, cover slipped as described above and imaged using a NikonA1plus confocal microscope. Cell counter ImageJ plugin was used to count tdT+ cells within lesions and cells in normal-appearing areas,namely: AT2 cells (LAMP3+), tdT+ AT2 cells (tdT+LAMP3+), AT1 cells (LAMP3+NKX2-1+, avoiding noticeable airways) and tdT+ AT1 cells (tdT+NKX2-1+LAMP3+). Percentages of tdT+LAMP3+ and tdT+NKX2-1+LAMP3+ cells out of total tdT+ cells were computed. Counts were averages of triplicate images taken at ×20 magnification for each time point. The percent regional surface area covered by tdT cells in normal-appearing regions was estimated by examining the tdT expression across entire lobe sections for each replicate.

3D culture and analysis of AT2-derived organoids
Gprc5a-/-;SftpcCreER/+;RosaSun1GFP/+ were treated with NNK or saline and tamoxifen as described above, and they were euthanized at EOE (4 saline-treated and 5 NNK-treated mice) or at 3 months after exposure (10 saline-treated and 13 NNK-treated mice). Lungs were collected, dissociated into single cells (see mouse single-cell derivation in the Methods section 'Single-cell isolation from tissuesamples'), and live (Sytox Blue-negative) GFP+ single cells were collected by flow cytometry using a FACS Aria I instrument as previously described. GFP+ AT2 cells from NNK-treated or saline-treated groups were immediately washed and resuspended at a concentration of 5,000 cells per 50 microliters of 3D medium (F12 medium supplemented with insulin, transferrin and selenium, 10% FBS,penicillin–streptomycin and l-glutamine). GFP+ cells were mixed at a 1:1 ratio (by volume) with 50,000 mouse endothelial cells (collected from mouse lungs by CD31 selection and expanded in vitro as previously described and resuspended in 50 microliters of Geltrex reduced growth factor basement membrane matrix (A1413301, Gibco). Next, 100 microliters of 1:1 GFP+:endothelial cell mixture was plated on Transwell inserts with 0.4 micrometer pores and allowed to solidify for 30 min in a humidified CO2 incubator (EOE: n = 3 wells per condition; 3 months after exposure: n = 4 wells for saline-derived organoids and n = 12 wells for NNK-derived organoids). Each well was then supplemented with 3D medium containing ROCK inhibitor (Y-27632, Millipore) and recombinant mouse FGF-10 (6224-FG, R&D Systems), and plates were incubated at 37 °C in a humidified CO2 incubator. Wells were replenished with 3D medium every other day. For GFP+ organoids derived from mice exposed to NNK, 200 nM KRAS(G12D)-specific inhibitor MRTX1133 or DMSO vehicle was added to the medium and replenished 3 times a week (n = 6 wells per condition). Organoids were monitored and analysed twice a week using an EVOS M7000 imaging system (Thermo Fisher Scientific), whereby the numbers and sizes of organoids greater than 100 micrometers in diameter were recorded. At end point, 3D organoids were collected from the basement membrane matrix using Gentle Cell Dissociation reagent(100-0485, StemCell Technologies), fixed with 4% paraformaldehyde, permeabilized, blocked and stained overnight at 4 °C with a mixture of IF primary antibodies raised against LAMP3, GFP, KRT8 and cavin 3. The next day, organoids were washed and stained with fluorophore-conjugated secondary antibodies overnight at 4 °C while being protected from light. Organoids were washed and stained with DAPI nuclear stain for 30 min, after which they were collected in Aqua-Poly/Mount (18606-20, Polysciences) and transferred to slides. Images of organoids were captured using an Andor Revolution XDi WD spinning disk confocal microscope and analysed using Imaris software (Oxford Instruments).

2D viability assays
Mouse mycoplasma-free LUAD cell lines LKR13 (mutant KrasG12D-driven) and MDA-F471 (Gprc5a-/- and KrasG12D mutant) were plated on 96-well plates (10 cells per well) and grown in DMEM (Gibco) supplemented with 10% FBS, 1% antibiotic antimycotic solution (A5955, Sigma-Aldrich) and 1% l-glutamine (G7513, Sigma-Aldrich). The next day, cells were cultured for up to 4 days with medium containing 0.5% FBS, 0.5% FBS with 50 ng/ml epidermal growth factor (EGF) (E5160, Sigma-Aldrich), or 0.5% FBS with EGF and varying concentrations of MRTX1133 (Mirati Therapeutics). alamarBlue Cell Viability reagent (25 microliters; DAL1025, ThermoFisher)was added to each well. At 4 days after treatment, viability was assessed by fluorescence spectrophotometry at 570 nm (and 600 nm as a reference). For the wells showing net positive absorbances relative to blank wells (at least 3 wells per cell line and condition), the percent differences in reduction between treated and control wells were calculated.

Western blot analysis
LKR13 and MDA-F471 cells were plated in 6-well plates (10 cells per well) and grown under different conditions as described above. Protein lysates were extracted at 3 h after treatment and analysed by western blotting following overnight incubation with antibodies tot he following primary proteins: vinculin (E1E9V, rabbit, Cell Signaling Technology, 13901; 1:1,000); phosphorylated p44/42 MAPK(ERK1/2, rabbit, Cell Signaling Technology, 9101; 1:2,000); phosphorylated S6 ribosomal protein (Ser 235/236, rabbit, Cell Signaling Technology, 4858; 1:2,000); p44/42 MAPK (ERK1/2, rabbit, Cell Signaling Technology, 9102; 1:2,000); or S6 (E.573.4, rabbit, Invitrogen, MA5-15164; 1:1,000). This was followed by 1 h of incubation with diluted secondary antibody (1706515 goat anti-rabbit IgG-HRP conjugate, Bio-Rad). Protein lysates from each cell line were analysed on multiple gels (four per cell line) with Precision Plus Protein Dual Color Standard (1610394, Bio-Rad) as the ladder and blotted to membranes to separately probe for phosphorylated and total forms of the same proteins, which have highly similar molecular weights (using phospho-specific antibodies or antibodies targeting total version of same protein). Vinculin protein levels were evaluated as loading control on each of the blots. Four blots (phospho-ERK, total ERK, phospho-S6 and total S6) for each of LKR13 and MDA-F471 are shown in Supplementary Fig. 9 each with its own analysis of equal protein loading (vinculin blot) and whereby only the ones indicated with green rectangles are presented in Extended Data Fig. 12c. Membranes were cut horizontally using molecular weight marker as a guide, and cut membranes were incubated with the specified antibodies (see Supplementary Fig. 9 for site of cutting and for overlay of colorimetric and chemiluminescent images of the same blot todisplay ladder and the analysed protein, respectively). Blots were imaged using the ChemiDoc Touch Imaging System (Bio-Rad) with Chemiluminescence and Colorimetric (for protein ladder) applications and auto expose or manual settings.

Statistical analyses
In addition to the algorithms and statistical analyses described above, all other basic statistical analyses were performed in the R statistical environment (v.4.0.0). The Kruskal–Wallis H -test was used to compare variables of interests across three or more groups. Wilcoxon rank-sum

test was used for paired comparisons among matched samples from the same patients. Wilcoxon rank-sum test was used to compare other continuous variables such as gene expression levels and signature scores between groups. Spearman's correlation coefficient was calculated to assess associations between two continuous variables (for example, cellular proportions and gene signature scores). Fisher'sexact test was used to identify differences in frequencies of groups based on two categorical variables. Ordinal logistic regression was performed using the polr function in the built-in R package MASS (v.7.3). Benjamin–Hochberg method was used to control for multiple hypothesis testing. All statistical tests performed in this study were two-sided. Results were considered significant at P values or FDR q values < 0.05. When a P value reported by R was smaller than 2.2e-16, it was reported as P < 2.2 × 10-16.

For manuscripts utilizing custom algorithms or software that are central to the research but not yet described in published literature, software must be made available to editors and reviewers. We strongly encourage code deposition in a community repository (e.g. GitHub). See the Nature Portfolio guidelines for submitting code & software for further information.

Data

Policy information about availability of data

All manuscripts must include a data availability statement. This statement should provide the following information, where applicable:

- Accession codes, unique identifiers, or web links for publicly available datasets
- A description of any restrictions on data availability
- For clinical datasets or third party data, please ensure that the statement adheres to our policy

Sequencing data for P1 - P5 were previously generated and deposited in the European Genome–phenome Archive (EGA) under the accession number EGAS0000100502115. Human scRNA-seq (P6 – P16) and ST data generated in this study have been deposited in EGA under the same accession number (EGAS00001005021). Mouse scRNA-seq and ST data generated in this study have been deposited in NCBI under GEO accession number GSE222901. Source data are provided with this paper.

Field-specific reporting

Please select the one below that is the best fit for your research. If you are not sure, read the appropriate sections before making your selection.

[x] Life sciences [ ] Behavioural & social sciences [ ] Ecological, evolutionary & environmental sciences

For a reference copy of the document with all sections, see nature.com/documents/nr-reporting-summary-flat.pdf

Life sciences study design

All studies must disclose on these points even when the disclosure is negative.

Sample size	No statistical methods were used to predetermine sample size. We performed single-cell RNA sequencing (scRNA-seq) on n = 63 tissue samples from n = 16 patients with early-stage lung adenocarcinomas, and performed spatial transcriptomics analysis on tumour sample from one patient in this cohort. We also performed whole exome sequencing on tumour and normal lung tissues and, when available, peripheral blood mononuclear cells from the same cohort (n = 64). We also analyzed by scRNA-seq lungs of n = 16 Gprc5a-/- mice at end of exposure or at 7 months post-exposure to tobacco carcinogen or control saline (4 mice each). Additionally, we analyzed three lung tissue of 2 mice from 7 months post-exposure to tobacco carcinogen by spatial transcriptomics analysis. Furthermore, we performed scRNA-seq on GFP+ cells isolated (by sorting) from n = 4 AT2 reporter mice at 3 months post-exposure to tobacco carcinogen or saline (2 mice per group). We also stained by IF, lungs of Gprc5a-/-; Krt8-creER;RosatdT/+ reporter mice exposed to NNK or control saline and tamoxifen for activation of Cre recombinase in Krt8-expressing cells, and which we analyzed immediately following labelling with tamoxifen (EOE, NNK n = 3, saline n = 2) or at 8-12 weeks post NNK (follow-up after EOE, n = 3).
Data exclusions	No data were excluded from this study
Replication	The findings of the KAC signature were observed in human scRNAseq cohort and then validated in 1) TCGA LUAD cohort with matched normal controls (n = 52), 2) the PROSPECT cohort (n = 45), 3) two published scRNA-seq datasets from studies interrogating KrasG12D-driven mouse models by scRNA-seq, 4) a dataset human AT2 cells with and without inducible KRASG12D expression, and 5) our previously reported bulk RNA-sequencing expression dataset comprised of cancer stem cell/stem cell-like progenitor cells, in the form of 3D spheres, and their parental MDA-F471 counterparts (grown in 2D).<br><br>For mouse scRNA-seq analysis experiments, we analyzed n = 4 Gprc5a-/- mice per treatment group and timepoint (16 mice in total), and n = 2 AT2 reporter mice per group (4 mice in total). The presence of KACs and the expression of KAC markers and/or KAC signature were validated in both human and mouse tissues by IF (n = 3 human LUAD cases, n = 4 for mouse) and by spatial transcriptomics analysis (n = 1 human LUAD tissue, n = 3 lungs from 2 mice). We also confirmed the presence of KACs in organoids derived from AT2 reporter mice at two timepoints, at the end of exposure (n = 4 or 5 mice at EOE to saline or NNK) or at 3 months post-exposure (n = 10 or 13 mice at 3 months post-saline or NNK, respectively), and in organoids with or without targeted KRAS inhibition in vitro. We also analyzed by IF Krt8 expression in tumour-bearing lungs of Krt8 reporter mice exposed to tobacco carcinogen (n = 6 mice, 17 total tumours), and compared to saline exposed animals (n = 2). All IF staining (tissue and organoids) were repeated at least 3 times with similar results. In vitro viability and western blot analyses were repeated two and three times, respectively, with similar results.
Randomization	This study does not involve samples from human subjects in clinical trials. This study utilized de-identified genomic and clinical data derived from patients undergoing surgical lung resection, and thus randomization does not apply to this study. In all animal studies, sex- and age-matched animals were randomized to treatment groups and analysis of mouse data was blinded.
Blinding	This study does not involve human subjects in clinical trials, blinding does not apply to this study.

Reporting for specific materials, systems and methods

We require information from authors about some types of materials, experimental systems and methods used in many studies. Here, indicate whether each material, system or method listed is relevant to your study. If you are not sure if a list item applies to your research, read the appropriate section before selecting a response.

Materials & experimental systems

n/a	Involved in the study
☐	☒ Antibodies
☐	☒ Eukaryotic cell lines
☒	☐ Palaeontology and archaeology
☐	☒ Animals and other organisms
☒	☐ Human research participants
☒	☐ Clinical data
☒	☐ Dual use research of concern

Methods

n/a	Involved in the study
☒	☐ ChIP-seq
☒	☐ Flow cytometry
☒	☐ MRI-based neuroimaging

Antibodies

Antibodies used	For sorting of epithelial cells: Human: EPCAM-PE (347198, BD Biosciences); Mouse: CD45-PE/Cy7 (BioLegend 103114), ICAM2-A647 (Life Technologies A15452), EPCAM-BV421 (Biolegend 118225), and ECAD-A488 (eBioscience 53-3249-80).<br><br>For immunofluorescence: LAMP3 (clone 391005, Synaptic Systems), KRT8 (TROMA-I clone from the University of Iowa DSHB), CLDN4 (ZMD.306 from Invitrogen), PRKCDBP (cavin3, Proteintech), GFP (Abcam, AB13970), PDPN (clone 8.1.1, University of Iowa DSHB), and NKX2-1 (sc-13040, Santa Cruz).<br><br>For digital spatial imaging: : Claudin 4 (clone 3E2C1, AF594, LSBio, catalog number LS-C354893 concentration 0.5 µg/ml), Keratin 8 (clone EP1628Y, AF647, Abcam, catalog number ab192468, concentration 0.25 µg/ml), PanCk (clone AE1/AE3, AF532, concentration 0.25 µg/ml, from GeoMx Solid Tumour Morp Kit HsP, 121300301, Novus Biologicals, Littleton, CO).
Validation	All antibodies were acquired from commercial vendors and used according the manufacturer's instructions. Antibody optimizations for digital spatial imaging of human tissues were performed with different dilutions using colorectal carcinoma and lung adenocarcinoma tissue.

Eukaryotic cell lines

Policy information about cell lines

Cell line source(s)	mouse
Authentication	MDA-F471 were established in house. We had developed and cultured MDA-F471 cells from a KM-LUAD of an NNK-exposed Gprc5a-/- mouse. LKR13 is a cell line that was previously derived from a tumour in the KrasLA1 model of KrasG12D-mutant LUAD (PMID: 15833854) and was a kind donation from Dr. Jonathan Kurie.
Mycoplasma contamination	These cells are routinely tested and validated to be free of mycoplasma contamination. Latest test was in July 2023 and is available upon request.
Commonly misidentified lines (See ICLAC register)	Cell lines used are not among the commonly misidentified cell lines.

Animals and other organisms

Policy information about studies involving animals; ARRIVE guidelines recommended for reporting animal research

Laboratory animals	All animals were Mus Musclus and with B6/SV129 mixed background. Krt8-creER (stock number 017947) and RosatdT/+ (Ai14; stock number 007914) mice were obtained from the Jackson Laboratory. SftpcCreER/+; RosaSun1GFP/+ mice were obtained from Dr. Harold Chapman (University of California, San Francisco). We studied three strains: Gprc5a-/- mice, Gprc5a-/-; SftpcCreER/+; RosaSun1GFP/+, and Gprc5a-/-;Krt8-creER;RosatdT/+ mice. Mice used in experiments were 8 weeks old and of both sexes.
Wild animals	This study did not involve wild animals.
Field-collected samples	This study did not involve animals collected from the field.
Ethics oversight	Mouse handling and care followed the NIH Guide for Care and Use of Laboratory Animals. All animal procedures followed the guidelines of and were approved by the MDACC Institutional Animal Care and Use Committee (IACUC protocol 00000800, PI: Kadara).

Note that full information on the approval of the study protocol must also be provided in the manuscript.
